# FastaValidator: an open-source Java library to parse and validate FASTA formatted sequences

**DOI:** 10.1186/1756-0500-7-365

**Published:** 2014-06-14

**Authors:** Jost Waldmann, Jan Gerken, Wolfgang Hankeln, Timmy Schweer, Frank Oliver Glöckner

**Affiliations:** 1 Microbial Genomics and Bioinformatics Research Group, Max Planck Institute for Marine Microbiology, Celsiusstrasse 1, 28359 Bremen, Germany; 2 Jacobs University Bremen gGmbH, Campusring 1, 28759 Bremen, Germany; 3 Mediomix GmbH, Eupener Straße 139, 50933 Köln, Germany

**Keywords:** FASTA, Data validation, High-throughput

## Abstract

**Background:**

Advances in sequencing technologies challenge the efficient importing and validation of FASTA formatted sequence data which is still a prerequisite for most bioinformatic tools and pipelines. Comparative analysis of commonly used Bio*-frameworks (BioPerl, BioJava and Biopython) shows that their scalability and accuracy is hampered.

**Findings:**

FastaValidator represents a platform-independent, standardized, light-weight software library written in the Java programming language. It targets computer scientists and bioinformaticians writing software which needs to parse quickly and accurately large amounts of sequence data. For end-users FastaValidator includes an interactive out-of-the-box validation of FASTA formatted files, as well as a non-interactive mode designed for high-throughput validation in software pipelines.

**Conclusions:**

The accuracy and performance of the FastaValidator library qualifies it for large data sets such as those commonly produced by massive parallel (NGS) technologies. It offers scientists a fast, accurate and standardized method for parsing and validating FASTA formatted sequence data.

## Findings

### Background

The introduction of the first DNA sequencing methods [1] established the discipline of bioinformatics with sequences as the primary source of data. With the advent of massive parallel “Next Generation Sequencing (NGS)” technologies [2] the speed of sequence production has now reached petabytes per year. The FASTA format was introduced alongside with the first algorithms and tools for biological sequence analysis [3,4]. It defines how sequences are formatted and exchanged in a simple human-readable layout. Today, the FASTA format is the de facto standard to exchange sequence data between bioinformatic tools. Several common frameworks exists offering FASTA sequence import and validation [5]. Concerning their functionality, many of these frameworks are rather complex and not designed for high-volume FASTA parsing and validation. Another common approach is the implementation of custom solutions. Often these have problems recognizing system-specific line endings (Unix, Microsoft, Apple), invalid characters, or even semantically incorrect data. This leads to serious problems in data processing up to invalid results. Furthermore, the focus of bioinformatics has shifted towards (web-based) pipelines that perform a range of consecutive tasks to analyze sequence data. Therefore, easy integration of FASTA import and validation functionality into larger software pipelines or workflows is becoming a common request. To address issues of parsing, validation, integration, scalability and performance, we present the light-weight, open-source FastaValidator library written in Java, which parses and validates sequences in FASTA format. The implementation in the platform-independent Java programming language assures broad usage and easy integration into bioinformatic software and pipelines. The performance of the library in comparison to state of the art frameworks has been evaluated and the ease of integration into web projects has been demonstrated.

### Implementation

The FastaValidator library implements the IUPAC specifications [6-8] extended by letters necessary to parse aligned sequences (space, dash, dot, asterisk). Based on these specifications four parsing modes are implemented: (1) A universal mode that parses and validates any (multi)FASTA file comprising the nucleotide and amino acid alphabets. (2) A DNA mode, which parses and validates only DNA nucleotide sequences. (3) An RNA mode, which parses and validates only RNA nucleotide sequences. (4) A Protein mode, which parses and validates only amino acid sequences. To implement the FastaValidator library for high performance, well established techniques from compiler construction have been used. A lexical analyzer (lexer) to parse and syntactically validate the FASTA format was generated using the JFlex scanner generator. The lexer first transforms all characters of a given FASTA file into syntactically correct tokens. The parsing mode defines the allowed characters accepted by the lexer. In a second step the correct semantic order of these tokens is validated (e.g. the header must be followed by a comment or sequence). If a FASTA file contains only correct tokens in the right order, it is valid. For every token (end of file (EOF), header-, comment- or sequence line) an event is generated and lines can be transformed into user defined data structures. To compile the FastaValidator from the source code Java 1.5 or higher, JFlex 1.4.3 or higher (http://www.jflex.de) and Ant 1.8 or higher (http://ant.apache.org) are required.

#### Performance tests

Automated evaluation tests were carried out on a standard Desktop-PC (Intel Core i5, 3 GHz, 16 GB RAM) running the 64 bit server version of Ubuntu Linux 12.04. For performance comparison all tests were run with BioJava 3.0.7 (http://biojava.org), Biopython 1.63 (http://biopython.org) and BioPerl 1.6.9 (http://www.bioperl.org). The underlying test environments were OpenJDK 1.7.0_25 for FastaValidator and BioJava, Python 2.7.3 and PyPy 2.2.1 for Biopython and Perl 5.14.2 for BioPerl.

Six different data sets were used as input data: (A) all protein sequences of *Escherichia coli* K-12 [9], (B) the complete genome of *Escherichia coli* K-12 [9], (C) all protein sequences of the SWISSPROT database as of December 2013 [10], (D) one metagenomic sequence set from a sampling site of the Global Ocean Sampling Expedition (JCVI_SMPL_1103283000001) [11], (E) the unaligned rRNA gene sequences of the SILVA database (SILVA release 115, SSU Parc) [12] and (F) the aligned sequences of the SILVA SSU reference database (SILVA release 115, SSU Ref NR) [12].

As test scenario the counting of valid letters in the input sequence data was chosen. This included the validation of the input data. Where necessary, the original parsers of the Bio*-Frameworks were extended by a few lines of code to perform this validation step based on the available letter alphabets of the respective frameworks. The overall constraint for these extensions was to keep the changes as minimal as possible to minimize the influence on the original performance. Each test was performed ten times. The test scripts as well as the raw results are available on the project’s website.

#### FastaValidatorUI

For end-users who do not intend to write their own software the FastaValidatorUI (User Interface) can be downloaded from the project website. It is a platform-independent Java application built on top of the FastaValidator library. With its two modes, command-line and graphical user interface, it can directly be used for high-throughput pipelines as well as for interactive validation without any knowledge in programming. The sources of FastaValidatorUI show how the FastaValidator library can be integrated in self-written tools. It is located in the demo directory of the FastaValidator source code repository.

### Results and discussion

**Figure 1:**
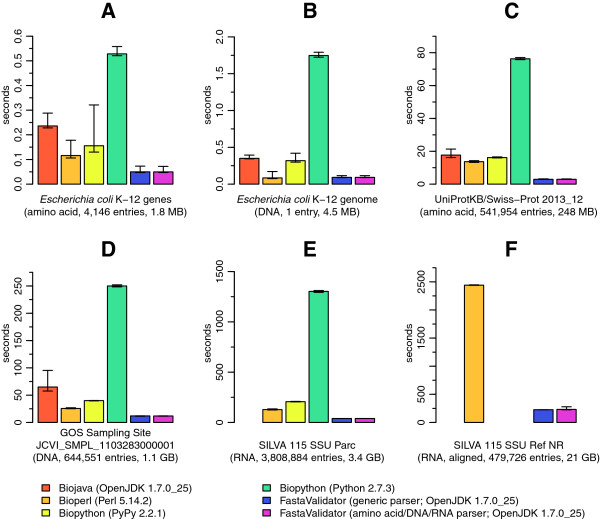
**Comparison of validation performance of three bioinformatic frameworks and FastaValidator.** Validation performance of three bioinformatic frameworks in comparison to FastaValidator with six different data sets. **(A)** all protein sequences of *Escherichia coli* K-12, **(B)** the complete genome of *Escherichia coli* K-12, **(C)** the protein sequences of the SWISSPROT database, **(D)** the metagenomic sequence set from the sampling site 1103283000001 of the Global Ocean Sampling Expedition, **(E)** the unaligned complete rRNA genes of the SILVA database and **(F)** the aligned sequences of the SILVA SSU reference database. Missing bars indicate that the corresponding test failed.

The results in Figure 1 show that the FastaValidator is on average the fastest validating parser and that it performs especially well on high-volume sequence data sets. Whilst the other frameworks tested have models for the different sequence letter alphabets in their design, but most of them did not use them properly in their implementations of the FASTA parser. Depending on the input sequence data the insufficient validation by these frameworks might finally lead to invalid sequences, which can cause serious problems in further downstream processing or even lead to wrong results. Aligned sequences could only be parsed successfully by BioPerl and FastaValidator, because the modeled alphabets of BioJava and Biopython are lacking dots which are commonly found in aligned sequences. Although not used for validation, some of the frameworks have the capability of auto detecting the alphabet, and by that, the type of an unknown input sequence. These methods cannot be considered as robust, because the amino acid and DNA letter alphabets have overlaps, especially when ambiguities are included.

### Conclusions

The accuracy and performance of the FastaValidator library qualifies it for large data sets as they are commonly produced by massive parallel (NGS) technologies. The ease of integrating FastaValidator into (web based) software pipeline and its efficiency is demonstrated in the open source project CDinFusion [13] and the SILVAngs high-throughput data analysis service for ribosomal RNA gene sequence data (https://www.arb-silva.de/ngs/). For end-users interested in validating their sequence data the ready to use FastaValidatorUI can be downloaded from the project’s website. In summary, FastaValidator offers scientists a fast, accurate and standardized method for parsing and validating FASTA formatted sequence data.

## Availability and requirements

**Project name:** FastaValidator.**Project home page:**http://www.megx.net/FastaValidator**Source code repository:**https://github.com/jwaldman/FastaValidator**Operating system(s):** Platform-independent.**Programming language:** Java.**Other requirements (pre-built):** Java 1.5 or higher.**Other requirements (build from scratch):** Java 1.5 or higher, JFlex 1.4.3 or higher, Ant 1.8 or higher.**License:** Lesser GPL 3 (LGPL 3).**Any restrictions to use by non-academics:** None.

## Competing interests

The authors declare that they have no competing interests.

## Authors’ contributions

JW and JG designed and implemented the software library as well as planning and execution of the performance tests. WH and JW drafted the manuscript. TS participated in the test script implementation and execution. FOG revised the manuscript critically. All authors read and approved the final manuscript.
